# AI Communication Tone and Consumer Judgment: The Role of Servant Perception in Behavioral Intentions

**DOI:** 10.3390/bs16020253

**Published:** 2026-02-10

**Authors:** John Yang

**Affiliations:** Business School, Hankuk University of Foreign Studies, Seoul 02450, Republic of Korea; johnyang@hufs.ac.kr

**Keywords:** human–AI interaction, social cognition, judgment, AI communication, tone, role construal, servant perception, agency attribution

## Abstract

Artificial intelligence is increasingly embedded in service interactions, requiring users to form rapid social judgments about AI communicators based on limited linguistic and contextual cues. This research examines how AI communication tone shapes behavioral intentions through social cognitive processes of role construal and agency attribution. Drawing on politeness theory, formality research, and social cognition perspectives, two scenario-based experiments test whether formal versus casual tone influences responses via attitudes toward the tone and the AI, and how these effects depend on perceptions of AI as a servant-like social actor. Study 1 shows that tone effects are moderated by servant perception and that economic framing, specifically paid versus free access, functions as an antecedent of hierarchical role construal. Study 2 replicates these effects and demonstrates that interaction structure, one-way versus two-way communication, similarly shapes servant perception by signaling differential autonomy. Across both studies, formal tone is more effective when AI is construed as subordinate, whereas casual tone is less effective under hierarchical role frames. By identifying servant perception as a central social cognitive mechanism, this research advances understanding of human judgment and decision making in technology-mediated interactions and offers implications for AI communication design aligned with role expectations. Because both studies rely on U.S. consumers, the findings should be interpreted within cultural contexts characterized by relatively low power distance, where role expectations and hierarchy norms may differ from other cultural settings.

## 1. Introduction

Artificial intelligence is increasingly embedded in everyday human decision making through frontline service interactions, where individuals must rapidly interpret social cues and infer intentions from minimal information. Across service and consumption contexts, conversational agents such as chatbots, kiosks, and service robots now routinely communicate with users using language that conveys not only information but also social meaning. Prior research shows that these interactions shape judgments of trust, competence, and appropriateness by activating social cognitive processes related to role expectations and interpersonal inference (Nass & Moon, 2000; Sundar & Nass, 2001). Complementing this perspective, a growing body of work suggests that AI-mediated communication fundamentally alters how people construe social roles and responsibility in technology-mediated encounters, thereby shaping evaluations and behavioral intentions across contexts (Belanche et al., 2020; I. Tussyadiah, 2020; Ivanov & Webster, 2019).

Despite extensive research on conversational agents, less is understood about how communication tone influences human judgment when individuals evaluate AI as a social actor rather than a neutral tool. Prior studies demonstrate that linguistic tone shapes perceptions of warmth, trust, and social presence in human–AI interaction, yet findings remain inconsistent across contexts, reflecting differences in social role framing and agent perception (Nass & Moon, 2000; Araujo, 2018). Social cognitive research suggests that such inconsistency arises because tone is interpreted through relational frames that specify expectations about roles, hierarchy, and appropriateness. For example, informal language can increase warmth and engagement in egalitarian settings, whereas formal language may signal respect and competence under hierarchical norms (Irvine, 1979; Morand, 1995). Accordingly, judgments of AI communication tone are likely to depend not only on the linguistic style itself but also on how users construe the relational role of the AI within the interaction (Brown & Levinson, 1987; Solomon et al., 1985).

A central mechanism underlying these effects is how individuals construe the social role of AI during interaction, particularly whether the AI is perceived as a subordinate or a social partner. Users frequently interpret conversational AI as occupying a servant-like role, applying hierarchical social schemas that position the system as subordinate rather than equal (Guzman, 2019; Schweitzer et al., 2019; Tschopp et al., 2023). Social cognitive research suggests that such role construals guide normative expectations about appropriate language, deference, and responsibility, thereby shaping evaluative judgments. Empirical evidence shows that servant-like framing influences perceptions of entitlement, trust, and compliance in human–AI interaction (Lei & Xie, 2025; Teng et al., 2024; Fiestas Lopez Guido et al., 2025; Kim & Kramer, 2015). Building on this work, the present study theorizes that servant perception functions as a key moderator in how communication tone is interpreted, such that formal language aligns with hierarchical role expectations under servant construal, whereas casual language becomes more effective when AI is perceived as a collaborative social actor (Solomon et al., 1985; Homburg et al., 2011, Lin et al., 2022; Eagly & Karau, 2002).

The present study further proposes that contextual cues embedded in AI system design shape social role inferences by signaling power, control, and relational orientation. Two cues are especially relevant for human judgment: whether access to the AI is framed as paid or free, and whether the interaction structure is one-way or two-way. Research on social power and dependence suggests that payment heightens hierarchical expectations by framing the AI as an entity under the user’s control or employment (French et al., 1959; Kwon & Jang, 2012; Kim & Kramer, 2015; Emerson, 1962). Similarly, interaction structure provides social cognitive signals about agency and reciprocity, with one-way communication emphasizing asymmetry and subordination, and two-way exchange fostering mutuality and perceived partnership (Grunig & Hunt, 1984; Men & Stacks, 2014; Guzman, 2019). Together, these contextual cues are expected to shape servant-like role construals, thereby conditioning how communication tone is evaluated and how judgments and decisions unfold in human–AI interaction (Eagly & Karau, 2002; Sundar, 2008).

This research develops and tests a social cognitive framework that explains how communication tone shapes human judgment and decision making in interactions with artificial intelligence. Although prior research has extensively examined linguistic tone in human–AI interaction, existing findings remain fragmented and often contradictory, with informal or social-oriented communication sometimes enhancing evaluations and at other times undermining perceived appropriateness or competence. These inconsistencies persist in part because prior work has largely treated communication tone as having uniform effects, without sufficiently accounting for how users construe the social role and agency of AI systems.

To address this limitation, the present research theorizes servant perception as a pre-existing role construal that moderates how communication tone is interpreted, rather than as an outcome of communication style itself. Specifically, the framework proposes that AI communication tone influences behavioral intention through a sequential evaluative process, in which attitudes toward the tone inform attitudes toward the AI, and ultimately guide decision making. This process is theorized to be contingent on how individuals construe the social role of the AI, with servant perception moderating the interpretation of linguistic cues. Two contextual cues, whether access to the AI is framed as paid or free and whether the interaction is structured as one-way or two-way, are examined as antecedents that shape servant-like role construals. Across two scenario-based experiments with U.S. consumers, the findings provide consistent support for this framework. Formal tone proves more effective when hierarchical role construals are salient, whereas casual tone is more effective when the AI is construed as a collaborative social actor. Together, these findings advance understanding of social cognitive mechanisms underlying role-based judgment and decision making in human–AI interaction (Eagly & Karau, 2002; Sundar, 2008).

## 2. Literature Review

### 2.1. Communication Orientation (Task vs. Social)

Communication orientation refers to the extent to which an interaction emphasizes task execution versus social affiliation, shaping how communicators are cognitively categorized and evaluated (Dabholkar et al., 2009; Verhagen et al., 2014). Originating from Bales’ (1950) Interaction Process Analysis, this distinction has been widely applied to explain how interaction styles guide interpersonal perception, attribution, and judgment across contexts (Figl & Saunders, 2011; Yao et al., 2022; Keeling et al., 2010; Wang et al., 2025). Social-oriented communication cues encourage perceivers to treat an agent as a social actor, thereby activating social cognitive processes such as mind attribution, warmth inference, and trust formation (Araujo, 2018; Von der Pütten et al., 2010; Verhagen et al., 2014; De Cicco et al., 2020). Consistent with this view, research shows that social-oriented styles enhance perceived warmth and social presence, which in turn influence evaluative judgments and behavioral intentions (Xu et al., 2022; Wang et al., 2025; Liebrecht et al., 2020; Cai et al., 2024).

Despite these insights, prior work has largely treated communication orientation as a direct driver of positive affect or trust, rather than as a cue that informs deeper role-based judgments. From a social cognitive perspective, interaction style does more than signal friendliness; it provides information about relational norms, role expectations, and appropriate conduct, which guide how linguistic cues are interpreted. Research on person perception and role inference suggests that identical behaviors can elicit divergent evaluations depending on whether the target is construed as subordinate, equal, or authoritative (Eagly & Karau, 2002; Fiske et al., 2007). Accordingly, the present research moves beyond the task–social dichotomy by focusing on formality and politeness as linguistic signals whose meaning depends on how the AI’s social role is construed, rather than assuming uniformly positive effects of social-oriented communication.

### 2.2. Politeness Theory

Politeness theory conceptualizes language use as a mechanism through which individuals manage social relations and make inferences about others’ intentions, respect, and relational stance. Brown and Levinson (1987) propose that communicators are motivated to protect face, defined as a socially valued self image, and that linguistic choices serve as cues for evaluating interpersonal consideration and normative appropriateness. From a social cognitive perspective, politeness strategies function as signals that inform judgments about how an actor understands and adheres to social norms, rather than merely mitigating face threatening acts (Brown & Levinson, 1987; Holtgraves, 1992). As such, politeness provides perceivers with diagnostic information for interpreting communicative intent and forming evaluative judgments.

Importantly, politeness judgments are context dependent and shaped by perceived power relations and role expectations. Brown and Levinson (1987) identify power, social distance, and imposition as key determinants of linguistic strategy selection, and subsequent work shows that these variables also guide how utterances are interpreted by observers (Holtgraves, 1992; Holtgraves & Yang, 1992). Social cognition research suggests that identical linguistic forms can be evaluated as respectful or inappropriate depending on whether they align with inferred role relations, such as subordinate versus equal actors (Eagly & Karau, 2002; Fiske et al., 2018). Accordingly, politeness theory is especially useful for understanding how communication tone shapes judgment and decision making when the communicator’s social role is ambiguous, as is often the case in interactions with artificial agents. From this perspective, the evaluative impact of AI communication tone is not inherent to the linguistic form itself but depends on how users construe the AI’s social role and associated power relations. Given such pre-existing role construals, communication tone is expected to influence attitudes toward the tone and the AI, with the magnitude and direction of these effects varying as a function of servant perception.

### 2.3. Formality

Formality refers to the degree of officialness or casualness in language, expressed through lexical choices, syntactic complexity, and colloquial markers (Heylighen & Dewaele, 2002). While politeness theory focuses on managing face concerns, formality highlights how linguistic style itself communicates social structure and role alignment, independent of specific speech acts (Irvine, 1979; Morand, 1995). Formal expressions tend to convey deference, authority, and normative appropriateness, whereas informal expressions signal relational closeness and reduced social distance. From a social cognitive perspective, formality operates as a cue that guides judgments about status, responsibility, and expected conduct, shaping how communicators are evaluated (Fiske et al., 2018; Eagly & Karau, 2002).

Prior research demonstrates that formality cues systematically influence evaluative judgments by activating competence and authority inferences. Studies across organizational and consumer contexts show that formal appearance and language increase perceived professionalism, reliability, and responsibility attribution, whereas informal cues are more likely to evoke warmth and approachability judgments (Morand, 1995; Yan et al., 2011; Shao et al., 2004; Ruetzler et al., 2012; C. Liu & Xie, 2023). Importantly, these effects are not uniform but depend on contextual expectations regarding roles and hierarchy, such that the same level of formality may be evaluated positively or negatively depending on whether it aligns with inferred social roles (Irvine, 1979; Smith et al., 2020).

Despite extensive evidence that formality shapes perception, most studies treat it as a direct stylistic determinant rather than as a socially diagnostic cue whose meaning depends on role construal. Building on social cognition and role congruity perspectives, the present research conceptualizes formality as a linguistic signal whose evaluative impact is contingent on how the communicator is socially categorized. When an actor is construed as subordinate, formality may be interpreted as appropriate and respectful, whereas informality may violate normative expectations. Accordingly, formality is theorized here as a conditional cue that links communication tone to judgment and decision making through role-based interpretation, providing a foundation for examining servant-like perceptions of artificial agents (Eagly & Karau, 2002; Sundar, 2008). Taken together, research on formality indicates that formal versus casual language serves as a socially diagnostic cue whose meaning is contingent on role expectations. When AI is construed as occupying a servant-like, subordinate role, formality is more likely to be interpreted as appropriate and respectful, whereas casual tone may violate hierarchical norms. Under such role construals, these role-contingent interpretations motivate the proposed mediation of tone effects through evaluative attitudes, with servant perception functioning as a moderating condition.

### 2.4. AI as Servant Perception

Beyond linguistic features such as politeness and formality, a central determinant of judgment in human–AI interaction is how individuals construe the AI’s social role. Research in social cognition demonstrates that people routinely categorize others into role-based schemas that guide expectations, responsibility attribution, and normative evaluation (Fiske et al., 2018; Eagly & Karau, 2002). Consistent with this perspective, studies on human–computer interaction show that users often construe artificial agents as subordinate social actors, applying interpersonal norms even in the absence of genuine intentionality (Nass & Moon, 2000; Guzman, 2019; Schweitzer et al., 2019). These findings suggest that servant-like role construals reflect a broader social cognitive tendency to interpret artificial agents through familiar human role categories. Accordingly, in this research, servant perception refers to the extent to which an AI system is socially construed as occupying a servant-like role, characterized by subordinate positioning under hierarchical role expectations, such that it is expected to execute user-directed goals rather than to engage as an autonomous or reciprocal social partner.

Empirical research indicates that servant-like role construals have systematic consequences for judgment and decision making. When an agent is perceived as subordinate, evaluations tend to be shaped by hierarchical expectations, including norms of deference, compliance, and appropriateness (Kim & Kramer, 2015; Teng et al., 2024). Such role-based construals influence how communicative behaviors are interpreted, often reducing perceived autonomy and altering responsibility attributions, which in turn shape trust, entitlement, and behavioral responses (Schweitzer et al., 2019; Lei & Xie, 2025). From a social cognitive standpoint, these effects arise because servant schemas activate assumptions about status, control, and acceptable conduct, thereby biasing evaluative judgments.

Importantly, servant-like role construals often emerge by default in interactions with artificial agents, even without explicit design cues. Research shows that conversational AI is frequently interpreted as a tool-like or command-following entity, with fewer users construing it as a partner or equal (Schweitzer et al., 2019; Tschopp et al., 2023). However, social cognition research emphasizes that the implications of a given role construal depend on contextual alignment between expectations and observed behavior (Fiske et al., 2018; Eagly & Karau, 2002; C. Liu & Xie, 2023). Accordingly, identical linguistic cues may be evaluated positively or negatively depending on whether they are perceived as congruent with a servant role or as a violation of hierarchical norms. Conceptually, servant perception is distinct from construing AI as a social partner, companion, or collaborator, because partner-like construals imply reciprocity and relational equality, whereas servant perception implies hierarchical role expectations in which authority and control are asymmetrically assigned to the user. This distinction determines how communicative cues, such as tone, are interpreted within the interaction.

Building on this logic, the present research theorizes servant perception as a key social cognitive moderator that conditions how communication tone is interpreted. When AI is construed as a servant, formal tone is expected to align with norms of deference and appropriateness, whereas casual tone may violate role expectations and elicit negative judgments. Thus, servant-like role construals provide a mechanism through which linguistic cues are translated into evaluative judgments and decision making outcomes, linking communication tone to attitudes and behavioral intention (Eagly & Karau, 2002; Sundar, 2008). Accordingly, the following hypotheses are advanced:

**H1.**  *AI communication tone (formal vs. casual) influences behavioral intention through a serial mediation path consisting of attitude toward the tone and attitude toward the AI.*

**H2.**  *The extent to which users perceive AI as a servant moderates the effect of communication tone (formal vs. casual) on tone attitude, such that formal tone becomes more favorable and casual tone less favorable when servant perception is high.*

### 2.5. Antecedents of AI as Servant Perception

From a social cognitive perspective, perceptions of AI as a servant do not arise solely from linguistic tone but are shaped by broader contextual cues that structure role expectations and agency attributions. Social cognition research shows that people rely on situational signals to infer others’ status, intentionality, and responsibility, especially when direct interpersonal cues are limited (Fiske et al., 2018; Weiner, 2000). In human–technology interactions, such inferences are particularly sensitive to cues that imply control, dependence, and asymmetry, which can lead users to construe AI as a subordinate social actor rather than an autonomous partner (Nass & Moon, 2000; Schweitzer et al., 2019).

One salient antecedent of servant perception is economic framing, such as whether access to AI is paid or free. From a social cognitive perspective, payment functions as a situational cue that structures perceived role relations by highlighting entitlement, obligation, and asymmetric dependence, rather than merely reflecting transaction cost. Social cognition research shows that perceived control and dependence shape role expectations by assigning asymmetric authority and responsibility within an interaction (French et al., 1959; Emerson, 1962; Weiner, 2000). When users pay for access to an AI system, they are more likely to construe the relationship as one in which the AI is accountable for executing user-directed goals under hierarchical role expectations. This framing activates service-related role schemas associated with compliance, deference, and instrumental responsibility, biasing users toward construing the AI as a servant-like, subordinate actor rather than as an autonomous or reciprocal partner. Prior consumer research similarly demonstrates that paid service arrangements heighten perceived entitlement and normative expectations regarding appropriate conduct, shaping evaluations even in the absence of human intentionality (Kim & Kramer, 2015). Accordingly, payment operates as a contextual signal that increases servant perception by emphasizing asymmetry in control and obligation, rather than partnership or mutual agency.

A second important antecedent is interaction structure, particularly whether communication is one-way or two-way. Social cognition theories emphasize that reciprocal interaction signals agency, intentionality, and mental states, whereas unilateral interaction promotes object-like or tool-based construals (Fiske et al., 2018; Waytz et al., 2010; Grunig & Hunt, 1984; Men & Stacks, 2014). In technology-mediated encounters, one-way communication emphasizes command-following and reduces perceived autonomy, thereby strengthening servant-like interpretations of AI. In contrast, two-way interaction encourages users to infer responsiveness and intentional engagement, mitigating hierarchical framing and supporting more partner-like role perceptions (Guzman, 2019; Tschopp et al., 2023; Go & Sundar, 2019).

**Figure 1:**
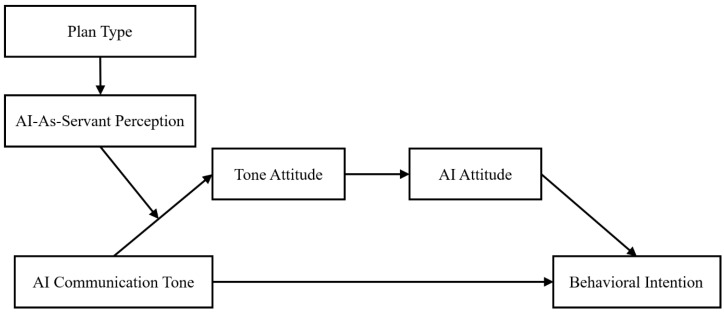
Conceptual Model with Plan Type as an Antecedent of Servant Perception.

**Figure 2:**
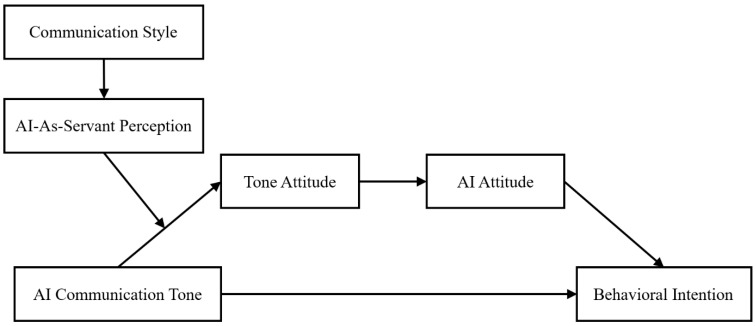
Conceptual Model with Communication Style as an Antecedent of Servant Perception.

Together, these contextual antecedents shape how users socially construe AI, providing the cognitive foundation upon which tone effects operate. By identifying payment structure and interaction design as antecedents of servant perception, this study situates AI role construal within broader social cognitive mechanisms of agency attribution and responsibility inference, extending prior research on human judgment and decision making in technology-mediated contexts (Fiske et al., 2018; Weiner, 2000). Based on this reasoning, the following hypotheses specify how plan type and communication style systematically shape AI-as-servant perceptions. The corresponding conceptual models are presented in Figure 1 and Figure 2.

**H3a.**  *Users are more likely to perceive AI as a servant when using a paid plan than when using a free plan.*

**H3b.**  *Users are more likely to perceive AI as a servant when interacting through one-way rather than two-way communication.*

## 3. Study 1

Study 1 examined how AI communication tone (formal vs. casual) shapes users’ social judgments and behavioral intentions by influencing attitudes toward the tone and the AI itself. Drawing on social cognition research suggesting that role expectations and agency attributions guide evaluations under conditions of limited interpersonal information (Fiske et al., 2018; Weiner, 2000), this study further tested whether these effects are moderated by perceptions of AI as a servant. In addition, plan type (paid vs. free) was examined as a contextual antecedent that structures hierarchical role construals in technology-mediated interactions (French et al., 1959; Kim & Kramer, 2015).

### 3.1. Participants

Participants were recruited via Prolific, using screening criteria to ensure they were U.S. residents whose first language was English and who were fluent in English. This linguistic control was critical because the study investigated fine-grained differences in tone perception. To avoid cultural or linguistic confounds, only native English speakers residing in the United States were included. This approach enhanced internal validity and ensured interpretive consistency for tone manipulations.

A total of 948 participants completed the survey and received £0.20. Participants were excluded if they spent less than 1.5 s per item (Bowling et al., 2023), had reCAPTCHA scores below 0.5 (Qualtrics, 2024), or showed extreme response times (Ratcliff, 1993). After exclusions, the final sample was 714 participants. The final sample had a mean age of 43.2 years (SD = 13.9) and 61.1% identified as female.

### 3.2. Method

The experiment used a 2 (plan type: paid vs. free) × 2 (tone: formal vs. casual) between-subjects design. Plan type was manipulated but not treated as a moderator. Instead, it was tested as an antecedent of servant perception.

Participants were randomly assigned to plan type: in the paid condition, the AI required a $20 monthly subscription (mirroring ChatGPT Plus Plan fees, version 5.2); in the free condition, it was available at no cost. Participants first read this plan type scenario and were instructed to imagine that they were about to use the AI under the described access condition. Within each condition, participants were randomly assigned to tone. In the formal tone condition, the AI greeting was: “Hello. How may I assist you?” In the casual tone condition, it was: “Hey! What’s up? What can I do for ya?” These greetings were selected to operationalize differences in linguistic formality and politeness, as formal expressions conventionally signal deference and role-appropriate professionalism, whereas casual expressions convey reduced social distance and informality (Brown & Levinson, 1987; Irvine, 1979; Morand, 1995; Heylighen & Dewaele, 2002). These greetings were designed to represent clear contrasts in formality while maintaining equivalent semantic content. The greeting was displayed in a standardized, screenshot-style chat interface to ensure consistent stimulus presentation across conditions. After viewing the greeting, participants completed the questionnaire measures.

Consistent with prior research on linguistic tone and politeness, the casual greeting was intentionally selected to reflect an informal, low-distance interaction style rather than to mirror standard professional service scripts. The purpose of this manipulation was not to approximate typical commercial phrasing but to create a theoretically meaningful contrast in perceived formality, allowing the study to isolate how linguistic informality is interpreted under different role construals. From a social cognitive perspective, such contrasts are essential for testing whether tone evaluations depend on pre-existing role expectations rather than on perceived realism alone. Such informal expressions are commonly used in conversational AI contexts that emphasize social presence or peer-like interaction, particularly in non-institutional or free-access settings, making the manipulation appropriate for testing role-contingent interpretations of tone.

### 3.3. Measures

All measures used a 7-point Likert scale (1 = strongly disagree, 7 = strongly agree).

Attitude toward the AI’s tone was assessed with three items (α = 0.96): “I really like this AI’s speaking style,” “I think this AI’s speaking style is really good,” and “my overall impression of this AI’s speaking style is very positive” (Silvera & Austad, 2004; De Liver et al., 2007).

Attitude toward the AI itself was measured with the same three items, adapted to refer to the AI rather than its tone (α = 0.97): “I really like this AI,” “I think this AI is really good,” and “my overall impression of this AI is very positive.”

Behavioral intention was captured with two items assessing intention to use the AI (α = 0.96): “If I need an AI, I would really like to use this one” and “if I need an AI, I would certainly choose this one” (Taylor & Baker, 1994).

Servant perception was measured with three items (α = 0.86): “This AI is very much like a friend or partner (1) versus a servant (7),” “this AI is very much like a servant,” and “this AI is very much like a friend, a partner, or a companion” (reverse-coded) (Kim & Kramer, 2015).

### 3.4. Results and Discussion

All analyses were conducted using SPSS (version 30.0.0.0). Manipulation checks and mean differences were examined using ANOVA. Hypothesized mediation and moderated mediation effects were tested using Hayes’ PROCESS macro (Models 6 and 83), with 5000 bootstrap samples to estimate bias-corrected confidence intervals. Effects were considered significant when the 95% confidence interval did not include zero.

An ANOVA manipulation check confirmed that participants in the formal condition rated the AI’s tone as more formal than those in the casual condition (M = 4.74 vs. 1.57; F(1, 712) = 1048.42, *p* < 0.001).

A serial mediation model (PROCESS Model 6; 5000 bootstraps; Hayes, 2017) supported H1. AI communication tone influenced behavioral intention indirectly through attitudes toward the tone and the AI (effect = 0.5177, 95% CI [0.3892, 0.6596]). Formal tone improved tone attitudes, which enhanced AI attitudes and, in turn, behavioral intention. The direct effect of tone on behavioral intention was not significant (95% CI [−0.1613, 0.0882]), indicating that the effect operated primarily through the mediation pathway.

A moderated serial mediation model (PROCESS Model 83; 5000 bootstrap samples; Hayes, 2017) supported H2. The sequential indirect effect of tone on behavioral intention via tone and AI attitudes was moderated by servant perception (index = 0.1453, 95% CI [0.0659, 0.2319]). Specifically, the conditional indirect effect was stronger when servant perception was high (effect = 0.7707, 95% CI [0.5632, 1.0021]) than when low (effect = 0.2863, 95% CI [0.1213, 0.4498]). Thus, formal tone became more effective under strong servant-role perceptions.

Lastly, plan type shaped servant perception, supporting H3a. Participants in the paid condition perceived the AI more as a servant than those in the free condition (M’s = 5.34 vs. 4.90; F(1, 712) = 16.57; *p* < 0.001), confirming its role as an antecedent of servant perception. Plan type had no direct effects on tone attitude, AI attitude, or behavioral intention, consistent with its role as an antecedent rather than a direct predictor.

Overall, Study 1 demonstrates that AI communication tone shapes behavioral intention through a social cognitive process in which users infer relational roles and agency from contextual cues. Consistent with research on role-based judgment and responsibility attribution (Fiske et al., 2018; Weiner, 2000), tone influenced behavioral intention indirectly via attitudes toward the tone and the AI, with servant perception moderating this pathway. Formal tone was advantageous when AI was construed as a subordinate social actor, whereas casual tone was less effective or even counterproductive under hierarchical role frames. Importantly, plan type functioned as a contextual antecedent that heightened servant perceptions by signaling control and dependence, thereby shaping how tone was socially interpreted (French et al., 1959; Kim & Kramer, 2015).

## 4. Study 2

Study 2 replicated and extended Study 1 by further examining how interaction structure shapes social judgments about AI communication. Building on social cognition research showing that reciprocity and interactivity are central cues for agency and intentionality attribution (Fiske et al., 2018; Waytz et al., 2010), this study tested whether communication style (one-way vs. two-way) functions as an antecedent of servant perception. By shifting attention from economic framing to interaction design, Study 2 assesses whether relational construals of AI generalize across distinct contextual cues that structure perceived hierarchy in technology-mediated encounters (Sundar & Nass, 2001; Guzman, 2019).

### 4.1. Participants

Recruitment was restricted to individuals who had not participated in Study 1. A total of 845 participants completed the survey via Prolific and received £0.20. Exclusion criteria matched Study 1: participants were removed if they spent less than 1.5 s per item (Bowling et al., 2023), had reCAPTCHA scores below 0.5 (Qualtrics, 2024), or showed extreme response times (Ratcliff, 1993). The final sample was 703 participants. The final sample had a mean age of 42.6 years (SD = 13.9) and 65.4% identified as female.

### 4.2. Method

The experiment employed a 2 (communication style: one-way vs. two-way) × 2 (tone: formal vs. casual) between-subjects design. Communication style was manipulated but not treated as a moderator. Instead, it was tested as an antecedent of servant perception.

In the one-way condition, participants were told that the AI robot followed instructions without questioning, giving opinions, or making its own decisions. In the two-way condition, they were told that the AI could ask questions, offer opinions, and sometimes propose alternatives. Participants first read the communication style scenario and were instructed to imagine interacting with the AI under the described interaction structure. As in Study 1, participants within each condition were randomly assigned to tone. In the formal tone condition, the AI greeting was: “Hello. How may I assist you?” In the casual tone condition, it was: “Hey! What’s up? What can I do for ya?” The greeting was presented using the same standardized interface format as in Study 1. After viewing the greeting, participants completed the questionnaire measures. Consistent with Study 1, this tone manipulation was designed to vary perceived formality rather than to approximate standard professional service language, allowing examination of how informal linguistic cues are interpreted under different assumptions about AI agency and interaction structure (Brown & Levinson, 1987; Irvine, 1979; Morand, 1995; Heylighen & Dewaele, 2002).

### 4.3. Measures

All measures used a 7-point Likert scale (1 = strongly disagree, 7 = strongly agree). The same measurement instruments as in Study 1 were used for attitude toward the AI’s tone (α = 0.96), attitude toward the AI itself (α = 0.96), intention to use (α = 0.96), and AI as servant perception (α = 0.93).

### 4.4. Results and Discussion

The same analytical procedures as in Study 1 were used. All analyses were conducted using SPSS. Manipulation checks and mean differences were examined using ANOVA, and hypothesized mediation and moderated mediation effects were tested using Hayes’ PROCESS macro (Models 6 and 83) with 5000 bootstrap samples. Statistical significance was determined based on 95% bias-corrected confidence intervals.

An ANOVA manipulation check confirmed that participants in the formal condition rated the AI’s tone as more formal than those in the casual condition (M’s = 5.45 vs. 1.51; F(1, 701) = 2063.69; *p* < 0.001).

A serial mediation model (PROCESS Model 6; 5000 bootstraps; Hayes, 2017) supported H1. AI communication tone influenced intention to use indirectly through attitudes toward the tone and the AI (effect = 0.4600, 95% CI [0.3375, 0.5898]). The direct effect of tone on intention to use was not significant, indicating that the effect operated primarily through mediation.

A moderated serial mediation model (PROCESS Model 83; 5000 bootstraps; Hayes, 2017) supported H2. The sequential indirect effect of tone on intention to use via tone and AI attitudes was moderated by servant perception (index = 0.1235, 95% CI [0.0502, 0.2001]). Specifically, the conditional indirect effect was stronger when servant perception was high (effect = 0.6518, 95% CI [0.4865, 0.8305]) than when low (effect = 0.2813, 95% CI [0.1215, 0.4469]). Thus, formal tone was more effective under strong servant-role perceptions.

Lastly, communication style shaped servant perception, supporting H3b. Participants in the one-way condition perceived the AI more as a servant than those in the two-way condition (M’s = 6.41 vs. 4.58; F(1, 701) = 376.60; *p* < 0.001), confirming its role as an antecedent of servant perception. Communication style had no direct effects on tone attitude, AI attitude, or intention to use, consistent with its role as an antecedent rather than a direct predictor.

Overall, Study 2 corroborates the proposed social cognitive framework by demonstrating that interaction design shapes how users construe AI roles and agency. Consistent with theories of mind perception and social judgment, one-way communication reduced perceived autonomy and reinforced servant-like construals, whereas two-way interaction mitigated hierarchical framing by signaling responsiveness and intentional engagement (Fiske et al., 2018; Waytz et al., 2010). As a result, communication style functioned as a key antecedent of servant perception that conditioned tone effects on attitudes and behavioral intention. These findings extend prior work by showing that relational interpretations of AI are not fixed but dynamically shaped by interaction structure in technology-mediated contexts.

## 5. General Discussion

Findings across two experiments demonstrate that the effects of AI communication tone depend critically on how users socially construe AI, particularly whether it is perceived as a servant-like social actor. Consistent with social cognition research on role inference and agency attribution, tone influenced behavioral intention indirectly through attitudes toward the tone and the AI, with this process moderated by servant perception (Fiske et al., 2018; Weiner, 2000). Contextual cues such as plan type and communication style systematically shaped these construals: paid plans and one-way interaction heightened servant framing, whereas free access and two-way exchange reduced hierarchical role interpretations. As a result, formal tone proved more effective under strong servant perceptions, while casual tone was less effective or even counterproductive. These findings help reconcile inconsistent results in prior research on conversational tone by showing that relational framing, rather than tone per se, determines whether formality or informality is advantageous (Nass & Moon, 2000; Schweitzer et al., 2019).

### 5.1. Theoretical Contribution and Managerial Implications

This research makes two key theoretical contributions. First, it extends the literature on communication tone in AI-mediated interactions by integrating communication orientation (task- vs. social-oriented), politeness theory, and formality with servant perception into a unified framework. Prior studies have highlighted that social-oriented or informal tone enhances warmth, rapport, and satisfaction in human–AI or human–robot interactions (Xu et al., 2022; De Cicco et al., 2020; Wang et al., 2025). The present findings demonstrate that the evaluative impact of communication tone depends critically on how users construe the social role of AI, rather than on tone alone, thereby advancing understanding of role expectations in guest–AI encounters (Chattaraman et al., 2019; C. Liu & Xie, 2023; Kumar et al., 2022).

Second, this study identifies contextual antecedents of servant perception in AI-mediated interactions. Study 1 showed that a paid subscription heightens hierarchical role framing, while Study 2 demonstrated that one-way communication elicits similar effects. These findings underscore that relational perceptions arise not only from tone but also from interactional and design features, contributing to research on contextual moderators in consumer–AI interaction (Kumar et al., 2022; van Pinxteren et al., 2023; Belanche et al., 2020).

For managers, the results suggest that casual tone may not always yield optimal outcomes. When AI is positioned as a subordinate tool, such as in subscription-based or command-oriented systems, a formal tone better aligns with user expectations and enhances evaluations. In contrast, when AI is framed as an autonomous partner, a casual tone can foster approachability and engagement without breaching role norms. Thus, managers should calibrate tone according to how the AI is positioned within the guest interaction journey. This strategic alignment can help enhance user satisfaction, trust, and willingness to engage with AI-driven systems and services (Lu et al., 2019; I. P. Tussyadiah et al., 2020).

### 5.2. Limitations and Future Research

Several limitations suggest avenues for future research. First, cultural norms regarding hierarchy, politeness, and power distance may moderate the observed effects, indicating the value of cross-cultural validation (Hofstede, 1984; Soares et al., 2007). Second, while this study focused on plan type and communication style as antecedents of servant perception, future work could examine additional design attributes such as anthropomorphism, affective expressiveness, or voice characteristics that may further recalibrate role-based social construals of AI (Araujo, 2018; Zhang et al., 2024; X. X. Liu et al., 2024; Shiramizu et al., 2022).

Relatedly, future research could connect the social cognitive mechanisms identified in this study to responsibility-based processes examined in AI-powered advertising and customization contexts. Prior work demonstrates that AI-driven ad customization influences behavioral intentions through psychological ownership and responsibility, with engagement shaping how customization translates into responsibility and downstream attitudes (Yang et al., 2025; Peck et al., 2021). Integrating these streams would help clarify whether responsibility inferences similarly emerge from communicative cues, such as tone and role construal, servant perception, and from content-level interventions, such as customization, within broader human–AI interaction frameworks.

Finally, although the present studies centered on retail and service-related contexts, extending this framework to other domains such as healthcare or education may reveal boundary conditions under which role congruity and servant perceptions function differently (Eagly & Karau, 2002; Wirtz et al., 2018; Belanche et al., 2020).

## 6. Conclusions

This research demonstrates that the effectiveness of AI communication tone depends fundamentally on how users socially construe AI roles and agency within technology-mediated interactions. Across two experiments, tone influenced behavioral intention indirectly through attitudes toward the tone and the AI, with servant perception functioning as a key moderator that structured responsibility and agency. Contextual cues such as plan type and communication style systematically shaped these role construals, determining when formal tone aligned with hierarchical expectations and when casual tone became incongruent or less effective. By integrating politeness, formality, and servant perception within a social cognitive framework, this study advances understanding of human judgment and decision making in consumer–AI interaction and offers guidance for designing AI communication strategies that align with users’ role expectations in service contexts.

## Data Availability

The raw data supporting the conclusions of this article will be made available by the authors on request.
